# Attention-Deficit/Hyperactivity Disorder Treatment Pattern and Its Associated Outcomes Among Children Referred to Psychiatry and Mental Health Services in Malaysia: Protocol for a Retrospective Cohort Study

**DOI:** 10.2196/89752

**Published:** 2026-10-29

**Authors:** Nor Asiah Muhamad, Ling Shiao Ling, Nur Hasnah Maamor, Suria Hussin, Tengku Puteri Nadiah Tengku Baharudin Shah, Nurul Hidayah Jamalluddin, Fatin Norhasny Leman, Nurul Syarbani Eliana Musa, Fariza Yahya, Norliza Chemi, Norni Abdullah, Norli Abdul Jabbar, Nurashikin Ibrahim

**Affiliations:** 1 Sector for Evidence-based Healthcare, National Institutes of Health, Ministry of Health Malaysia, Jalan Setia Murni U13/52 Persiaran Setia Murni, Shah Alam, Selangor, 40170, Malaysia, 60 3-3362-8888 ext 8705; 2 Department of Psychiatry and Mental Health, Hospital Tengku Permaisuri Norashikin, Ministry of Health Malaysia, Kajang, Selangor, Malaysia; 3 Department of Psychiatry and Mental Health, Hospital Raja Perempuan Zainab II, Ministry of Health, Malaysia, Kota Bharu, Kelantan, Malaysia; 4 Research and Grant Management Unit, National Institutes of Health, Ministry of Health Malaysia, Shah Alam, Selangor, Malaysia; 5 Department of Psychiatry and Mental Health, Hospital Tengku Ampuan Rahimah, Ministry of Health Malaysia, Klang, Selangor, Malaysia; 6 National Center of Excellent Mental Health (NCEMH), Ministry of Health Malaysia, Cyberjaya, Selangor, Malaysia

**Keywords:** ADHD, hyperactivity, treatment, children, psychiatry, Malaysia

## Abstract

**Background:**

Attention-deficit/hyperactivity disorder (ADHD) is a common neurodevelopmental disorder associated with significant impairments in academic, social, and daily functioning. Despite the availability of clinical practice guidelines in Malaysia, evidence on real-world treatment patterns and outcomes remains limited. The lack of multicenter data on treatment use and clinical outcomes hinders the evaluation of current ADHD care and opportunities for service improvement.

**Objective:**

The primary objective of this study is to observe treatment patterns among children with ADHD referred to psychiatry and mental health services in Malaysia after receiving treatment for 18 months. This study will also compare treatment outcomes between children with ADHD who do receive treatment and those who do not receive any ADHD treatment.

**Methods:**

This multicenter retrospective cohort study will use secondary data from clinical records of children aged 4 to 16 years with a confirmed diagnosis of ADHD. Data on sociodemographic characteristics, clinical features, ADHD subtype and comorbidities, treatment modalities, health care use, treatment trajectories, and clinical outcomes will be extracted using a standardized case report form and will be anonymized prior to analysis. Data collection will be undertaken by trained site investigators and medical officers across participating centers. Statistical analyses will be conducted using R software (R Foundation for Statistical Computing). Descriptive statistics will be used to summarize characteristics, treatment patterns, health care use, and clinical outcomes of children with ADHD. Treatment patterns will be characterized according to pharmacological and nonpharmacological interventions, treatment modifications, and service use. Comparative analyses will be performed to examine associations between treatment patterns and clinical outcomes across children with ADHD and clinical subgroups.

**Results:**

This study will be funded through a Ministry of Health Research Grant obtained in August 2026. It will commence in August 2026 and end in December 2028. Based on the sample size calculation, a minimum of 2771 eligible children with ADHD records will be included in the study. The study will be reported in accordance with the STROBE (Strengthening the Reporting of Observational Studies in Epidemiology) guidelines for retrospective cohort studies. The findings will be disseminated through presentations and article publications at the national and international levels.

**Conclusions:**

This study will address a critical evidence gap in ADHD care by providing comprehensive real-world data on treatment patterns and outcomes among Malaysian children with ADHD. Findings will identify variations in treatment approaches, treatment trajectories, and clinical outcomes across hospitals and regions, assess the alignment of real-world practice with national recommendations, and highlight potential disparities in access to ADHD specialist services. The findings are expected to support evidence-based clinical decision-making, guide strategic health care planning, optimize resource use, and contribute to the development of a more coordinated, efficient, and equitable system of ADHD care across diverse health care settings in Malaysia.

## Introduction

Attention-deficit/hyperactivity disorder (ADHD) is one of the common neurodevelopmental disorders that are typically first diagnosed in childhood. ADHD is defined as persistent symptoms of inattention, hyperactivity, and impulsivity that may significantly interfere with daily functioning and development. Globally, the prevalence of ADHD among children aged 3 to 12 years and adolescents aged 12 to 18 years is 7.6% and 5.6%, respectively [1]. In Malaysia, 1.61% of primary school students aged 6 to 12 years are diagnosed with ADHD, with male children observed as having a higher prevalence compared to female children at a ratio of 4:1 [2]. Additionally, the prevalence of ADHD cases among children has increased from 4.6% in 2015 [3] to 7.1% in 2023 [4].

According to the *DSM-5* (*Diagnostic and Statistical Manual of Mental Disorders* [Fifth Edition]), ADHD is divided into 3 subtypes: attention deficit (ADHD-I), impulsive (ADHD-H), and mixed type (ADHD-C) [5]. Children and adolescents diagnosed with ADHD-I often may fail to pay close attention to details or may make careless mistakes [6], often have difficulty in sustaining attention when completing tasks, do not seem to listen when spoken to directly most of the time, often do not follow instructions, fail to finish tasks, have difficulty organizing tasks and activities, avoid eye contact most of the time, dislike or are reluctant to engage in tasks that require sustained mental effort, often lose things necessary for tasks or activities, are easily distracted by extraneous stimuli, and are often forgetful during daily activities [5]. Meanwhile, children and adolescents diagnosed with ADHD-H often fidget with or tap their hands or feet [6] or squirm in their seat [7], often leave their seat in situations when remaining seated is expected, often run about or climb in situations when it is inappropriate, often are unable to play or engage in leisure activities quietly, often are “on the go” [8], acting as if “driven by a motor,” often talk excessively, often blurt out an answer before a question has been completed, often have difficulty waiting their turn, and often interrupt or intrude on others.

To be diagnosed with ADHD, children up to age 16 must exhibit at least 6 symptoms within a specific domain or at least 6 from each domain for the combined presentation, and these symptoms must have emerged prior to age 12. These symptoms must persist for at least 6 months, be present in two or more settings (such as at school and at home), and cause clear evidence of functional impairment [5]. In addition, these symptoms must not occur exclusively during the course of schizophrenia or another psychotic disorder, and they must not be better explained by another mental disorder [5].

The occurrence of ADHD in children and adolescents is usually associated with genetic factors. The heritability of ADHD in a family is approximately 74%, and familial aggregation of ADHD increases with genetic relatedness [9,10]. The presence of genetic variants may lead to a risk of ADHD. In fact, in one study, it was found that 33% of children with ADHD had a parent with ADHD, and the child showed more severe ADHD than the parent [11]. However, the heritability of ADHD cannot be explained by genetic variants alone; it is reported to be due to gene-gene interaction [12], gene-environment interaction [13], and gene-environment correlation [14]. Environmental factors, such as prepregnancy maternal obesity, prenatal factors, and perinatal factors, may also contribute to the development or severity of ADHD among children and adolescents. Studies have shown evidence that maternal smoking during pregnancy, maternal obesity during prepregnancy, and maternal psychosocial stress increased the possibility of the offspring to be diagnosed with ADHD [15-17]. Persistent exposure to environmental stress may interrupt the neurodevelopment of offspring. Additionally, psychosocial factors like traumatic brain injury, the child’s diet, and the amount of screen time may also worsen the condition’s symptoms [18-20].

Globally, the main approaches to ADHD treatment can be categorized into pharmacological and nonpharmacological interventions. Nonpharmacological methods include occupational therapy, organizational skills training, and psychological interventions. Although these strategies have demonstrated effectiveness, for example, improving functional outcomes, enhancing sustained attention, reducing distractibility, and decreasing impulsive behaviors, there is evidence that the combination of pharmacological and nonpharmacological treatments yields the most effective results in managing ADHD symptoms and improving overall functioning [21].

In Malaysia, the number of children aged 5 to 15 years diagnosed with hyperactivity has increased from 4.6% in 2015 [3] to 7.1% in 2023; this is a worrying issue, as hyperactivity might contribute to ADHD [4]. A study by Sahril et al [22] found that younger age, single parents, parents working in the private sector, lower parental education, and parental mental health problems were major factors associated with mental health problems among children in Malaysia [4].

A clinical practice guideline (CPG) has been established, and several recommendations for the treatment of ADHD in children and adolescents are listed [23]. The purpose of these treatments is to improve the child’s ADHD clinical symptoms, functioning, academic achievements, medication adherence, and self-worth. Interventions include psychoeducation and pharmacological and nonpharmacological treatments. Nonpharmacological treatments include occupational therapy and psychological interventions such as cognitive behavioral therapy and family-based intervention (parent training and behavioral intervention). A previous study showed that cognitive behavioral therapy was effective for adolescents with ADHD who continued to exhibit persistent symptoms despite being prescribed medication [23,24]. The CPG recommends that pharmacological treatment, such as stimulants and nonstimulants, be offered to children aged 6 years or older and to adolescents. Nonstimulants that have Food and Drug Administration (FDA) approval for use in ADHD are atomoxetine, guanfacine, and clonidine. Other nonstimulants such as bupropion and modafinil have been shown to be superior to the placebo in reducing ADHD core symptoms [23].

Most children with ADHD who receive treatment show improvement in academic learning, clinical symptoms, and behavior with intervention. In Malaysia, pharmacological treatments such as stimulant medication (methylphenidate) and nonstimulant medication (atomoxetine) have been used to treat ADHD among children [25]. However, regarding nonpharmacological treatment, including psychoeducation, parent training, and occupational and behavior therapy [26], there is limited access or these options are unavailable to children in most hospitals in Malaysia due to cost, service, and accessibility constraints [3]. On a separate note, several treatments used internationally, such as amphetamine, dextroamphetamine, clonidine, digital therapy, school-based interventions, and electroencephalogram neurofeedback (a noninvasive technique that tracks real-time brainwave activity to teach self-regulation of the central nervous system), are currently unavailable in Malaysia. This gap may stem from variations in health systems and local resource availability across different countries [27], even though the clinical effectiveness of medications such as amphetamine has been widely documented [28].

Furthermore, there are no reports or data available that provide evidence on which is the most effective treatment for ADHD among children in Malaysia. A previous report stated that the combination of pharmacological and nonpharmacological treatments might be effective [3]. In short, the limited research on ADHD treatment patterns among children poses a challenge to clinicians, patients, and caregivers. Furthermore, despite existing guidelines for the management of ADHD in Malaysia, currently, no study has specifically observed treatment trends and outcomes in the local context. There are also inconsistent findings on the treatment pattern in ADHD among children globally due to local formulary restrictions, service capacity, and affordability, among other reasons [26]. On a separate note, there is an increasing number of clinicians practicing in this field, and standardized guidelines may help in guiding clinicians in the management of ADHD. The 2021 CPG on ADHD among children provides valuable but general guidance on management; consequently, this study is necessary to transition from general principles to a standardized, nationwide protocol. By evaluating current clinical practices, this study generates localized, living evidence to drive continuous improvement in care equity and delivery, ensuring that children in all states receive an identical, high standard of evidence-based ADHD management.

This study aims to address these gaps by determining ADHD treatment patterns and outcomes among Malaysian children by providing preliminary data to improve diagnosis, management, and interventions. This study will help clinicians and doctors understand real-world practice (actual clinical practice) and compare it to treatment guidelines. Treatment patterns include types of intervention received upon diagnosis of ADHD (pharmacological, nonpharmacological, or both), any changes in treatment over the follow-up period, and reasons for the changes. In addition, treatment outcomes using pharmacological and nonpharmacological interventions will evaluate the effectiveness of the intervention based on symptoms before and after the intervention. Findings will also evaluate the outcome based on the number of symptoms, a reduction or decrease in severity (evaluated by a clinician during a clinic session), functionality at home or in a social environment (subjective or objective report from caregiver/parent), and functionality at school (academic results or conduct in school) based on verbal reports from a schoolteacher or school results/formal school reports based on formal reports. All outcomes, such as academic improvement, decrease in symptoms, and functionality at home or in a social environment, will be recorded using the Clinical Global Impression-Improvement (CGI-I) scale. On a separate note, this study will also observe the current treatment trends received and their outcomes among children from the first date of diagnosis. This is important for improving the diagnosis, management, and treatment of ADHD among children.

The primary objective of this study is to observe treatment patterns among children with ADHD referred to psychiatry and mental health services in Malaysia after receiving treatment for 18 months. This study will also observe how the academic performance and ADHD symptoms and behaviors in children may improve or not improve at the 18-month follow-up. This study will also compare the treatment outcomes between children with ADHD who receive treatment and those who do not receive any ADHD treatment.

## Methods

### Study Design

This is a multicenter retrospective study using secondary data. It will be conducted by reviewing the children with ADHD to examine current treatment trends and outcomes among children with ADHD referred to psychiatry and mental health services in Malaysia.

### Study Population and Setting

This multicenter retrospective cohort study will be conducted among children and adolescents with ADHD receiving care through psychiatric and mental health services at 17 government hospitals across Malaysia. Eligible patients will be identified from medical records dated between January 1, 2021, and December 31, 2025, with study activities conducted from August 1, 2026, to December 31, 2028.

The participating hospitals were selected because they serve as major referral centers for psychiatric and mental health services in their respective states, providing broad geographical coverage across Malaysia. These centers are staffed by resident or regularly visiting child and adolescent psychiatrists and offer multidisciplinary ADHD management services, including pharmacological treatment, occupational therapy, speech and language therapy, parent management training, psychological interventions, and social skills programs. The inclusion of hospitals from all regions of the country is expected to provide a nationally representative overview of real-world ADHD treatment practices and outcomes within the Malaysian public health care system. A list of participating hospitals is provided in Multimedia Appendix 1.

### Eligibility Criteria

Patients will be eligible for inclusion if they are between the ages of 4 and 16 years at the time of ADHD diagnosis, have a documented clinical diagnosis of ADHD, are Malaysian citizens, and have received assessment or treatment through psychiatric and mental health services at one of the participating hospitals during the study period.

The lower age limit of 4 years was selected because ADHD can be reliably assessed and diagnosed during the preschool years, consistent with international CPGs. The upper age limit of 16 years was chosen to allow completion of the planned 18-month follow-up period before participants reach adulthood and transition to adult mental health services, which operate under different clinical pathways and models of care.

Only Malaysian citizens will be included to ensure a relatively homogeneous study population who have similar access to publicly funded health care services and treatment pathways. This criterion supports the study objectives of evaluating treatment patterns and outcomes within the Malaysian public health care system and generating evidence relevant to national health care planning and policy development.

Patients will be excluded if they are younger than 4 years or older than 16 years at the time of diagnosis, do not have a documented diagnosis of ADHD, are not Malaysian citizens, or have insufficient clinical information to determine treatment exposure or treatment outcomes during the follow-up period.

Patients with co-occurring psychiatric, neurodevelopmental, or medical conditions will not be excluded. Common comorbidities, such as autism spectrum disorder, anxiety disorders, depressive disorders, oppositional defiant disorder, conduct disorder, learning disorders, intellectual disability, and tic disorders, frequently coexist with ADHD and may influence treatment selection and clinical outcomes. Information on these comorbidities will therefore be extracted from medical records and included in the analysis as potential confounding or effect-modifying variables. Including patients with comorbid conditions is intended to reflect routine clinical practice and enhance the generalizability of the study findings.

All patient information will be deidentified prior to analysis and handled in accordance with institutional and ethical requirements. Study data will be stored securely in both electronic and hard-copy formats, with access restricted to authorized members of the research team.

### Recruitment and Sampling

This study will include children with ADHD who have complete clinical data available at baseline and at 6-, 12-, and 18-month follow-up assessments. An 18-month observation period was selected to allow sufficient time for treatment initiation, dose optimization, and evaluation of sustained treatment response in routine clinical practice. Previous studies have demonstrated that clinically meaningful improvements following pharmacological and behavioral interventions for ADHD are typically observable within the first 3 to 12 months of treatment, with longer follow-up periods enabling assessment of the durability of treatment effects [29,30].

A universal consecutive sampling approach will be used. All medical records meeting the predefined inclusion and exclusion criteria during the study period will be included regardless of whether the minimum sample size requirement has been met. This approach is appropriate for retrospective cohort studies as it minimizes selection bias, reflects real-world clinical practice, and enhances the representativeness of the study population across participating centers. Furthermore, inclusion of all eligible records increases statistical precision and enables evaluation of variations in treatment practices and outcomes across hospitals.

### Outcome Assessment

Treatment outcomes will be assessed using the Clinical Global Impression-Improvement (CGI-I) scale. The CGI-I is a widely used clinician-rated global measure that evaluates overall improvement relative to baseline status. As a single-item measure, internal consistency reliability (eg, Cronbach α) is not applicable. For the purposes of this study, treatment response will be categorized based on CGI-I ratings, with patients who are rated as “much improved” or “very much improved” considered as responders [31].

The 18-month follow-up period is expected to provide adequate time for assessment of treatment initiation, dose titration, completion of psychological interventions, and evaluation of sustained clinical improvement.

### Data Collection

This multicenter study will use a retrospective medical record review design. Data will be extracted from existing patient records at participating hospitals using a standardized data collection form developed by the research team. Information collected will include sociodemographic characteristics, ADHD subtype, psychiatric and medical comorbidities, treatment modalities, follow-up information, and documented clinical outcomes.

Prior to study commencement, all site principal investigators (PIs) will participate in a structured training program to ensure a standardized understanding of the study protocol, eligibility criteria, outcome assessment procedures, and data abstraction methods. Site PIs will also review the data collection form and provide feedback to enhance consistency across participating centers.

The site PIs, who are child and adolescent psychiatry specialists or clinicians directly involved in ADHD management, will identify eligible cases and extract relevant information from medical records. No additional patient interviews, assessments, investigations, or follow-up visits will be conducted specifically for this study.

In addition to data abstraction, site PIs will assign a CGI-I outcome rating based on their review of the documented clinical information available throughout the follow-up period. This rating represents a retrospective clinician assessment derived from existing clinical documentation rather than prospective collection of new patient-level data. Guidance on CGI-I scoring will be provided during investigator training to promote consistency across study sites.

### Pilot Study

Prior to the main study, a pilot study will be conducted at two participating hospitals: Hospital Tengku Permaisuri Norashikin, Selangor, and Hospital Raja Perempuan Zainab II, Kelantan. Approximately 30 children with ADHD records (15 records per hospital) will be reviewed to assess the feasibility of data extraction procedures, determine the availability of required variables within medical records, and refine the data collection process where necessary.

### Data Collection Procedures and Quality Assurance

Following investigator training, the PI and two research psychiatrists will conduct site visits to all participating hospitals to screen medical records for eligibility. Before each visit, the coordinating team will liaise with the site PI to arrange access to records and prepare a list of potentially eligible ADHD cases.

Eligible records will be identified and documented by the research team. Site PIs and designated medical officers will subsequently complete the standardized data collection forms using information extracted from the medical records. Completed forms will be submitted to the National Institutes of Health (NIH), Malaysia, where trained research personnel will enter the data into a secure electronic database.

To ensure data quality, regular data verification and monitoring procedures will be undertaken. Queries arising during data entry will be referred to the relevant site PI for clarification. In addition, investigator meetings will be conducted throughout the study to address operational issues and ensure consistent implementation of study procedures across centers.

Stakeholder engagement sessions will be conducted before study commencement and upon completion of the study to facilitate communication of study objectives, progress, and key findings to relevant stakeholders.

### Sample Size

The sample size was calculated using a proportion estimation formula with Epitools online software [32]. This is an independent cohort study that compares the event rates between exposed (patients who receive any ADHD treatment) and nonexposed (patients who do not receive any ADHD treatment) groups using 2 proportions. The percentage of expected incidence in the unexposed group is 30.1% [33]. The calculation is based on an assumed relative risk of 1.77 [34] with a precision of 0.05. With a 95% CI and 20% nonresponse rate, this study requires a minimum sample of 163 respondents. Therefore, with 17 participating hospitals, the minimum overall sample size required is 2771 patients.

### Research Tool

This data collection form consists of 6 sections, including (A) sociodemographic profile; (B) ADHD assessment and diagnosis; (C) medical history and comorbidities; (D) ADHD treatments at baseline, 6 months, 12 months, and 18 months; (E) monitoring and follow-up at baseline, 6 months, 12 months, and 18 months; and (F) treatment outcomes at 6 months, 12 months, and at 18 months. For sections D and E, there will be 4 sets of data collection, as the data will be collected at baseline, 6 months, 12 months, and 18 months. For section F, there will be three sets of data collection, at 6 months, 12 months, and 18 months. The collection form that will be used in this study is included in Multimedia Appendix 2.

#### Section A: Sociodemographic Characteristics

This section will collect general information on the respondent’s age, gender, ethnicity, religion, highest level of parental education, and household income status.

#### Section B: ADHD Diagnosis

This section will collect information on the age of onset, date of diagnosis, type of diagnosed ADHD, core symptoms of ADHD, and ADHD rating scale score.

#### Section C: Medical History and Comorbidities

This section will collect information on medical history, comorbidities, and any treatments, including the dates they were received, during the 90 days before ADHD treatment begins.

#### Section D: ADHD Treatment, Monitoring, Follow-Up, and Treatment Changes

This section will collect information on the type of initial treatment received by the child or adolescent with ADHD during the 18 months of the study period; this may include psychoeducation, nonpharmacological treatment, pharmacological treatment, and combination treatment. Information on treatment characteristics will be recorded, including dose; frequency; duration of treatment; changes in dose, frequency, or treatment and reasons for the changes; and treatment discontinuation date and reason.

#### Section E: Treatment Outcome

This section will collect information on changes in ADHD symptoms, functionality, treatment response, and academic performance based on rating scales available in the medical chart, formal reports, and clinician notes. In this study, changes in ADHD symptoms, academic performance, and functionality will be recorded using the CGI-I scale for ADHD in children [35]. This scale is a clinician-rated, single-item measure used to evaluate overall improvement or deterioration in a child’s ADHD symptoms after starting treatment (medication, behavioral therapy, or combined interventions). It facilitates practical clinical assessment by evaluating changes in a child’s overall functioning across home, school, and social settings. The scale consists of 7 rating categories, with lower scores indicating greater clinical improvement. Details on the CGI-I score are listed in Table 1. Prior to data collection, the PIs will provide training to site investigators and designated officers involved in data collection on the proper administration and completion of the CGI-I scale.

**Table 1.** Clinical Global Impression-Improvement score.

Score	Indicator
1	Very much improved
2	Much improved
3	Minimally improved
4	No change
5	Minimally worse
6	Much worse
7	Very much worse

This score will reflect overall changes in ADHD symptoms and functionality across environments. The clinician will compare participants’ current functioning with the baseline or pretreatment evaluation. The assessment and scoring of children with ADHD using this instrument involves a 3-step process conducted by clinicians. Step 1 involves integrating multiple sources. The score will be based on parent interviews, teacher reports, clinical observation, school performance, and the child’s behavior at home and during sessions. Step 2 involves comparing the child’s current behavior to the baseline. In this step, clinicians will observe attention or distractibility, hyperactivity or fidgeting, impulsiveness, school behavior, social interactions, and emotional regulation. Step 3 involves assigning the child a score using standard clinical criteria as listed in Table 2.

**Table 2.** Scoring of Clinical Global Impression-Improvement (CGI-I) scale using standard clinical criteria. Scores of 1-2 will be classified as improved (responder), scores of 3 as minimally improved (partial responder), and scores of 4 or more as not improved (nonresponder).

Score	Indicator	Criteria
1	Very much improved	Clear improvement, symptoms reduced to mild or near-normal, parents and teachers report major functional improvement, no major impairment remains, and behavior noticeably different
2	Much improved	Significant clinical improvement, symptoms clearly better but not fully normalized, most core ADHD problems have decreased and there is functional improvement at less than two places as reported by parents and teachers, for example, at home and school
3	Minimally improved	Small but noticeable improvement, symptoms slightly less severe, limited or inconsistent changes in functional and improvement only reported by either teachers or parents in one place or setting
4	No change	Similar symptoms appear during the baseline and no improvement in functionality
5	Minimally worse	Mild increase in hyperactivity, impulsiveness, or inattention and slight deterioration compared to the baseline
6	Much worse	Clear clinical worsening and functioning decrease in multiple places or settings
7	Very much worse	Deterioration appears and severe worsening of behaviors and symptoms

### Statistical Analysis

Data will be analyzed using the R statistical software environment (R Foundation for Statistical Computing). Statistical significance will be set at a 2-sided *P* value of <.05. Treatment pattern refers to the type of treatment received by children with ADHD, including pharmacological treatment, nonpharmacological treatment, and combined treatment approaches. Treatment outcome refers to treatment effectiveness as reflected by improvements in ADHD symptoms, behavioral functioning, academic performance, treatment adherence, and other clinically relevant indicators assessed in this study.

Descriptive statistics will be used to summarize participants’ sociodemographic and clinical characteristics, including age, sex, ethnicity, ADHD subtype, psychiatric and medical comorbidities, treatment modalities, and duration of follow-up. Categorical variables will be presented as frequencies and percentages, whereas continuous variables will be summarized using means and SDs or medians and IQRs, as appropriate.

Treatment patterns will be described according to the type of intervention received, including pharmacological treatment, nonpharmacological treatment, and combined treatment approaches. The distribution of treatment modalities across participating hospitals will also be reported.

Clinical outcomes will be evaluated using the CGI-I scale. Patients rated as “very much improved” or “much improved” will be classified as responders, whereas those rated as “minimally improved,” “no change,” or “worse” will be classified as nonresponders. The proportions of responders and nonresponders will be reported overall and by treatment modality. Associations between treatment patterns and treatment outcomes will be examined using the appropriate statistical tests. Comparisons of treatment response across treatment modalities will be conducted using the *χ*^2^ test or Fisher exact test, as appropriate.

To identify factors associated with treatment response, bivariable analyses will first be performed using the *χ*^2^ test or Fisher exact test for categorical variables and the independent *t* test or Mann-Whitney *U* test for continuous variables, as appropriate. Variables assessed will include age, sex, ethnicity, ADHD subtype, psychiatric comorbidities, neurodevelopmental comorbidities, treatment modality, and duration of treatment. Variables with a *P* value <.25 in the bivariable analyses, together with variables considered clinically relevant based on existing evidence, will be included in a multivariable logistic regression model. Treatment response (responder vs nonresponder based on CGI-I classification) will serve as the dependent variable. Adjusted odds ratios with 95% CIs will be reported to identify factors independently associated with treatment response.

### Missing Data

Data completeness will be assessed during data abstraction through verification against source medical records. Where feasible, missing information will be cross-checked with the original records and clarified with site investigators. The extent and pattern of missing data will be examined and reported for all study variables. As only patients with complete baseline, 6-month, 12-month, and 18-month follow-up data will be eligible for inclusion, missing outcome data are expected to be minimal. For variables with minimal missingness (<5%), complete-case analysis will be performed. Where substantial missing data are identified for explanatory variables, the pattern and mechanism of missingness will be evaluated, and multiple imputation may be considered if appropriate. Sensitivity analyses will be conducted where necessary to assess the potential impact of missing data on study findings.

### Ethical Considerations

This study had been registered in the National Medical Research Register (NMRR), and ethical approval had also been obtained from the Medical Research Ethics Committee of the Ministry of Health Malaysia (NMRR ID-25‐03130-ILL, investigator-initiated research [IIR]; see Multimedia Appendix 3), as well as relevant local authorities, prior to the start of data collection. Since this study will obtain patients’ data from medical records, the ethics approval requires that permission be obtained prior to the start of the study from the relevant district health officer, hospital administrator, or hospital director and the heads of departments or units where the study will be carried out. This study will be conducted under the close supervision of a trained medical officer on-site, ensuring absolute compliance with the ethical principles outlined in the Declaration of Helsinki and the Malaysian Guidelines for Good Clinical Practice. As this study will use secondary data collected by reviewing patients’ medical records, no informed consent from patients is needed. Data such as patients’ hospital ID will be anonymized for patient privacy and confidentiality.

## Results

This study was funded through a Ministry of Health Research Grant in August 2026 and will commence in August 2026 and end in December 2028, encompassing study preparation, pilot testing, and data collection. Data analysis and data management, including data cleaning, statistical analysis, and manuscript writing, will start in September 2028. Table 3 shows the project timeline for this study.

Participant identification and data abstraction are scheduled to commence in August 2026. Based on the sample size calculation, a minimum of 2771 eligible patient records will be included in the study.

The study will be reported in accordance with the STROBE (Strengthening the Reporting of Observational Studies in Epidemiology) guidelines for retrospective cohort studies. Statistical analyses will be conducted using the R statistical software environment.

The findings will be disseminated through presentations at national and international scientific conferences and publication in peer-reviewed journals. Manuscript submission is anticipated by January 2029.

**Table 3.** Project timeline.

Activities	Timeline
Pilot test	August 2026 to October 2026
Study briefing with all principal investigators at sites/hospitals	October 2026 to November 2026
Data collection	November 2026 to October 2027
Data cleaning	October 2027 to March 2028
Data analysis	March 2028 to September 2028
Manuscript writing	September 2028 to December 2028

## Discussion

### Principal Findings

Children with ADHD are reported to struggle with daily functioning, academic performance, and mental health. Usually, ADHD symptoms from childhood can last long into adulthood, which leads to a reduction in quality of life. This protocol describes a multicenter retrospective cohort study designed to generate comprehensive evidence on real-world treatment patterns and outcomes among children with ADHD receiving care within Malaysian psychiatric and mental health services. Despite the availability of national CPGs for ADHD among children, substantial variations in service delivery, treatment initiation, and multidisciplinary involvement are expected across facilities due to differences in resource allocation, provider training, and local clinical workflows [36,37]. By using standardized data extraction procedures and reviewing clinical records across multiple hospitals, this study is positioned to generate one of the first large-scale national datasets capturing ADHD management in Malaysia’s public health care system. Such evidence is essential in contexts where treatment pathways may be influenced by resource limitations, clinician training, and differences in access to specialist services [38].

The focus on treatment outcomes provides an opportunity to better understand the effectiveness of current practices in real-world settings. Previous research has highlighted that multimodal interventions tend to produce better functional and behavioral improvements than single-modality treatments [39,40]. However, real-world outcomes may be influenced by comorbidities, adherence issues, caregiver capacity, and service accessibility—factors often underrepresented in controlled trials [40]. By examining longitudinal data from diagnosis onward, this study is positioned to identify predictors of treatment response, persistence of symptoms, and service use patterns among Malaysian children. Such insights are expected to inform more targeted and contextually relevant clinical decision-making.

Overall, the evidence generated from this study is anticipated to support the refinement of national ADHD care pathways, support an update to the current CPG for ADHD among children, and guide the prioritization of clinical resources. By understanding treatment variability and associated outcomes, developing standardized treatment strategies for ADHD among children, and bridging gaps between guideline recommendations and actual practice, this study ultimately aims to contribute to the improvement of developmental, academic, and psychosocial trajectories for children with ADHD.

### Expected Outcomes and Impact

This study is expected to provide the first comprehensive national overview of treatment patterns and clinical outcomes among children with ADHD receiving care within psychiatric and mental health services in Malaysia. The findings will generate real-world evidence on the use of pharmacological and nonpharmacological interventions, treatment effectiveness, and factors associated with clinical response.

The study will provide important information on treatment outcomes based on clinician-rated improvement, including changes in ADHD symptoms, behavioral functioning, academic performance, and overall clinical status. In addition, the study will describe the occurrence of treatment-related adverse effects documented in routine clinical practice and evaluate the effectiveness of current therapeutic approaches across different health care settings. In short, significant associations between treatment patterns (type and modality of treatment received) and treatment outcomes (clinical and functional improvements among children with ADHD) are expected to be observed.

The findings are expected to support efforts to optimize and standardize ADHD management in the Malaysian public health care system. By identifying current treatment practices and associated outcomes, this study may inform future service planning, resource allocation, and the development or introduction of additional evidence-based treatment options where gaps in care are identified.

Furthermore, the findings will provide locally generated evidence to support future updates of the Clinical Practice Guidelines: Management of Attention Deficit Hyperactivity Disorder in Children and Adolescents [41]. The results may contribute to strengthening standardized, evidence-based care pathways and improving consistency in clinical practice across health care institutions.

Given the increasing prevalence and service demand associated with ADHD in Malaysia, the findings will also be relevant to policymakers, health care providers, educators, and other stakeholders involved in child development and mental health services. The evidence generated may facilitate intersectoral collaboration, particularly with the Ministry of Education Malaysia, to promote earlier identification, timely intervention, and improved support for children with ADHD across health care and educational settings.

### Limitations

This retrospective cohort study has several inherent limitations. The use of secondary data from clinical records may result in incomplete or missing information, which could affect the accuracy and comprehensiveness of the findings. Variability in documentation practices across multiple hospitals may introduce measurement bias. Additionally, the observational design limits the ability to establish causal relationships between treatment patterns and outcomes. Potential confounding factors, such as socioeconomic status or adherence to treatment, may not be fully captured in the records. Finally, the study population is limited to children referred to psychiatric and mental health services, which may reduce the generalizability of findings to all children with ADHD in Malaysia.

### Conclusions

This study protocol outlines a comprehensive approach to evaluating treatment patterns and outcomes among children with ADHD in Malaysia using a multicenter retrospective design. The findings are expected to provide valuable insights into current clinical practices, identify gaps in care, and inform evidence-based strategies for standardizing treatment. Ultimately, this study aims to support improved clinical decision-making, guide policy development, and contribute to better mental health outcomes for children with ADHD in Malaysia.

## Supplementary material

10.2196/89752 Multimedia Appendix 1 List of selected hospitals.

10.2196/89752 Multimedia Appendix 2 Data collection form.

10.2196/89752 Multimedia Appendix 3 Ethics approval letter.

## Abbreviations

ADHD: attention-deficit/hyperactivity disorder
ADHD-C: attention-deficit/hyperactivity disorder—combined
ADHD-H: attention-deficit/hyperactivity disorder—hyperactivity
ADHD-I: attention-deficit/hyperactivity disorder—inattention
CGI-I scale: Clinical Global Impression-Improvement Scal
CPG: clinical practice guideline
FDA: Food and Drug Administration
NIH: National Institutes of Health
NMRR: National Medical Research Registry
PI: principal investigator
STROBE: Strengthening the Reporting of Observational Studies in Epidemiology
