# Supplementary material for: Attention-Deficit/Hyperactivity Disorder Treatment Pattern and Its Associated Outcomes Among Children Referred to Psychiatry and Mental Health Services in Malaysia: Protocol for a Retrospective Cohort Study
Source: JMIR Res Protoc. 2026 Oct 29;15:e89752. doi: 10.2196/89752 (PMC13718534; doi:10.2196/89752)
Supplement: Supplementary file 1 [file resprot-v15-e89752-s003.pdf]

JAWATANKUASA ETIKA & PENYELIDIKAN PERUBATAN
(*Medical Research & Ethics Committee*)
KEMENTERIAN KESIHATAN MALAYSIA
d/a Kompleks Institut Kesihatan Negara
Blok A, No 1, Jalan Setia Murni U13/52,
Seksyen U13, Bandar Setia Alam,
40170 Shah Alam, Selangor.
Tel: 03-3362 8888/8205

Ref : 25-03130-ILL (2)
Date: 13-March-2026

DR. LING SHIAO LING
HOSPITAL TENGKU PERMAISURI NORASHIKIN, KAJANG

Dear Sir/ Mdm,

ETHICS INITIAL APPROVAL: NMRR ID-25-03130-ILL (IIR)
Attention Deficit Hyperactivity Disorder (ADHD) Treatment Pattern and Its Associated Outcomes among Children Referred to Psychiatric and Mental Health Services in Malaysia.

This letter is made in reference to the above matter.

2. The Medical Research and Ethics Committee (MREC), Ministry of Health Malaysia (MOH) has provided ethical approval for this study. Please take note that all records and data are to be kept strictly **CONFIDENTIAL** and can only be used for the purpose of this study. All precautions are to be taken to maintain data confidentiality. **Permission from the District Health Officer / Hospital Administrator / Hospital Director and all relevant heads of departments / units where the study will be carried out must be obtained prior to the study.** You are required to follow and comply with their decision and all other relevant regulations.

3. The investigators and study sites involved in this study are:

HOSPITAL KAJANG
Ling Shiao Ling (Principal Investigator)
Nor Azwani Binti Che Yusoff

HOSPITAL RAJA PEREMPUAN ZAINAB II
Suria Hussin (Principal Investigator)

Hospital Tunku Azizah (Hospital Wanita dan Kanak-Kanak Kuala Lumpur)
Anita Codati (Principal Investigator)

HOSPITAL SELAYANG
Nazariah Aiza Binti Harun (Principal Investigator)

HOSPITAL TENGKU AMPUAN RAHIMAH KLANG
Wan Asyikin Binti Wan Azlan (Principal Investigator)

HOSPITAL TENGKU AMPUAN AFZAN, KUANTAN
Noorul Amilin Harun (Principal Investigator)

HOSPITAL SULTANAH NUR ZAHIRAH, KUALA TERENGGANU
Wan Hanie Rasyida Binti Wan Hisham (Principal Investigator)

HOSPITAL TUANKU FAUZIAH, KANGAR
Ruzita binti Jamaluddin (Principal Investigator)

HOSPITAL RAJA PERMAISURI BAINUN, IPOH
Michelle Chan Su Ann (Principal Investigator)

HOSPITAL BAHAGIA ULU KINTA
Farah binti Ahmad Shahabuddin (Principal Investigator)

HOSPITAL SULTANAH BAHIYAH, ALOR SETAR
Yusni Yusuff (Principal Investigator)

HOSPITAL PULAU PINANG
Wong Yim Chan (Principal Investigator)

HOSPITAL TUANKU JAAFAR, SEREMBAN
Punitha A/P Udaya Kumar (Principal Investigator)

HOSPITAL MELAKA
Nurulwafa Hussain (Principal Investigator)

HOSPITAL PERMAI
Nor Rahidah Abd Rahim (Principal Investigator)

HOSPITAL UMUM SARAWAK
Ng Boon Seng (Principal Investigator)

HOSPITAL MESRA BUKIT PADANG
Ahmad Abdul Jalil Bin Mohd Razali (Principal Investigator)

NATIONAL INSTITUTES OF HEALTH (NIH)
Nur Hasnah Binti Ma'amor (Principal Investigator)
Nurul Syarbani Eliana binti Musa

4. The following study documents have been received and reviewed with reference to the above study:

Documents received and reviewed with reference to the above study:

1. Study Protocol_Version 7.0, dated 16-Feb-2026
2. Investigator's documents : Declaration of Conflict of Interest (COI), IA-HOD-IA, and CV:
   a) Ling Shiao Ling (Principal Investigator)
   b) Nor Azwani Binti Che Yusoff
   c) Suria Hussin (Principal Investigator)
   d) Anita Codati (Principal Investigator)
   e) Nazariah Aiza Binti Harun (Principal Investigator)
   f) Wan Asyikin Binti Wan Azlan (Principal Investigator)
   g) Noorul Amilin Harun (Principal Investigator)
   h) Wan Hanie Rasyida Binti Wan Hisham (Principal Investigator)
   i) Ruzita binti Jamaluddin (Principal Investigator)
   j) Michelle Chan Su Ann (Principal Investigator)
   k) Farah binti Ahmad Shahabuddin (Principal Investigator)
   l) Yusni Yusuff (Principal Investigator)
   m) Wong Yim Chan (Principal Investigator)
   n) Punitha A/P Udaya Kumar (Principal Investigator)
   o) Nurulwafa Hussain (Principal Investigator)
   p) Nor Rahidah Abd Rahim (Principal Investigator)
   q) Ng Boon Seng (Principal Investigator)
   r) Ahmad Abdul Jalil Bin Mohd Razali (Principal Investigator)
   s) Nur Hasnah Binti Ma'amor (Principal Investigator)
   t) Nurul Syarbani Eliana binti Musa

5. Please note that ethical approval is valid until **12-March-2027**. The following are to be reported upon receiving ethical approval. Required forms can be obtained from the National Medical Research Regisry (NMRR) website:

i. **Continuing Review Form** has to be submitted to MREC within 2 month (60 days) prior to the expiry of ethical approval.
ii. **Study Final Report** upon study completion to the MREC.
iii. Ethical approval is required in the case of **amendments / changes** to the **study documents/ study sites/ study team**. MREC reserves the right to withdraw ethical approval if changes to study documents are not completely declared.

6. This study involves the following methods:

    i. Retrospective
    ii. Medical records

7. Please take note that the reference number for this letter must be stated in all correspondence related to this study to facilitate the process.

Comments (if any): NIL

Project Sites:
HOSPITAL KAJANG
HOSPITAL RAJA PEREMPUAN ZAINAB II
Hospital Tunku Azizah (Hospital Wanita dan Kanak-Kanak Kuala Lumpur)
HOSPITAL SELAYANG
HOSPITAL TENGKU AMPUAN RAHIMAH KLANG
HOSPITAL TENGKU AMPUAN AFZAN, KUANTAN
HOSPITAL SULTANAH NUR ZAHIRAH, KUALA TERENGGANU
HOSPITAL TUANKU FAUZIAH, KANGAR
HOSPITAL RAJA PERMAISURI BAINUN, IPOH
HOSPITAL BAHAGIA ULU KINTA
HOSPITAL SULTANAH BAHIYAH, ALOR SETAR
HOSPITAL PULAU PINANG
HOSPITAL TUANKU JAAFAR, SEREMBAN
HOSPITAL MELAKA
HOSPITAL PERMAI
HOSPITAL UMUM SARAWAK
HOSPITAL MESRA BUKIT PADANG
NATIONAL INSTITUTES OF HEALTH (NIH)

Decision by Medical Research & Ethics Committee:
(√) Approved
(   ) Disapproved

Date of Approval: 13-March-2026

DR NURAIN MOHD NOOR
Chairperson
Medical Research & Ethics Committee
Ministry of Health Malaysia
MMC No: 31576
