# Supplementary material for: Attention-Deficit/Hyperactivity Disorder Treatment Pattern and Its Associated Outcomes Among Children Referred to Psychiatry and Mental Health Services in Malaysia: Protocol for a Retrospective Cohort Study
Source: JMIR Res Protoc. 2026 Oct 29;15:e89752. doi: 10.2196/89752 (PMC13718534; doi:10.2196/89752)
Supplement: Supplementary file 2 [file resprot-v15-e89752-s001.docx]

**APPENDIX**

**Appendix 1**

List of the selected hospital

1.	Hospital Tengku Permaisuri Norashikin, Selangor

2.	Hospital Raja Perempuan Zainab II, Kelantan

3.	Hospital Tunku Azizah, Kuala Lumpur

4.	Hospital Selayang, Selangor

5.	Hospital Tengku Ampuan Rahimah, Selangor

6.	Hospital Tengku Ampuan Afzan, Pahang

7.	Hospital Sultanah Nur Zahirah, Terengganu

8.	Hospital Tuanku Fauziah, Perlis

9.	Hospital Raja Permaisuri Bainun, Perak

10.	Hospital Bahagia Ulu Kinta, Perak

11.	Hospital Sultanah Bahiyah, Kedah

12.	Hospital Pulau Pinang, Pulau Pinang

13.	Hospital Tuanku Ja’afar, Negeri Sembilan

14.	Hospital Melaka, Melaka

15.	Hospital Permai, Johor

16.	Hospital Umum Sarawak

17.	Hospital Mesra Bukit Padang, Sabah
