# Supplementary material for: Attention-Deficit/Hyperactivity Disorder Treatment Pattern and Its Associated Outcomes Among Children Referred to Psychiatry and Mental Health Services in Malaysia: Protocol for a Retrospective Cohort Study
Source: JMIR Res Protoc. 2026 Oct 29;15:e89752. doi: 10.2196/89752 (PMC13718534; doi:10.2196/89752)
Supplement: Supplementary file 3 [file resprot-v15-e89752-s002.docx]

**Appendix 2**

## **Collection Form**

1. **Subject ID: __________________**
2. **Hospital:**

| - Hospital Kajang, Selangor | - Hospital Tuanku Fauziah, Perlis |
| --- | --- |
| - Hospital Tunku Azizah, Kuala Lumpur | - Hospital Sultanah Bahiyah, Kedah |
| - Hospital Permai, Johor | - Hospital Pulau Pinang, Pulau Pinang |
| - Hospital Sultanah Aminah, Johor | - Hospital Tuanku Ja’afar, Negeri Sembilan |
| - Hospital Selayang, Selangor | - Hospital Melaka, Melaka |
| - Hospital Ulu Kinta, Perak | - Hospital Raja Perempuan Zainab II, Kelantan |
| - Hospital Tengku Ampuan Rahimah, Selangor | - Hospital Raja Pemaisuri Bainun, Perak |
| - Hospital Tengku Ampuan Afzan, Pahang | - Hospital Umum Sarawak, Sarawak |
| - Hospital Sultanah Nur Zahirah, Terengganu | - Hospital Mesra Bukit Padang, Sabah |

**3. Source of referral:**

- Primary care (KK/ GPs)

- Paediatrics Department
- Others. Please specify: ________________

8.10

**Section A: Sociodemographic Data**

1. **Gender:**

| - Male | - Female |
| --- | --- |

2. **Age (Years) first seen at current hospital: _______________**
3. **Date of Birth: _______________________**
4. **Ethnicity:**

- Malay

- Chinese

- Indian

- Bumiputras Sabah & Sarawak

- Others (Please Specify): ________________

5. **School:**

- Mainstream (private/government)

- Program Pendidikan Khas Integrasi (PPKI)

- PDK (Pusat Pemulihan Dalam Komuniti)

6. **Academic Achievement:**

- Poor

- Below average

- Average
- Above average

- Not Attending School

7. **Number of Siblings:**

- 0
- 1-5

- ≥ 6

8. **Approximate Household Income based on family employment type:**

- Low household income/ B40 (< RM 4,850)

- Middle household income/ M40 (RM 4,851 – RM 10,970)

- High household income/ T20 (RM 10,971 and above)

- Insufficient information available

9. **Family Profile:**

**9.1. Father’s Age when first seen in current hospital:**

- <20
- 20 – 40

- ≥ 41

- Not stated

**9.2. Mother’s Age when first seen in current hospital:**

- ≤45

- > 45

- Not stated

**9.3. Father’s Educational Level:**

- Primary

- Secondary

- Tertiary

- No formal Education

- Not stated

**9.4. Mother’s Educational Level:**

- Primary

- Secondary

- Tertiary

- No formal Education

- Not stated

**9.5. Parent’s Marital Status:**

- Married

- Divorced

**9.6. Family members who are also diagnosed with ADHD**

- Yes, if yes, please state: ____________________

- No

**9.7. Any perinatal complications:**

- Yes. If yes, please state: __________________

- No

**Section B: ADHD Assessment and Diagnosis**

1. **Approximate age of Onset for ADHD (Years):** ___________________
2. **Type of diagnosed ADHD:**

- Hyperactive

- Inattentive

- Combined

- Insufficient information available

3. **ADHD Core Symptoms (Based on DSM-5th Edition):**

**Inattention (6 or more symptoms):**

- Often fails to give close attention to details or makes careless mistakes in schoolwork, at work, or during other activities (e.g., overlooks or misses details, work is inaccurate).

- Often has difficulty sustaining attention in tasks or play activities (e.g., has difficulty remaining focused during lectures, conversations, or lengthy reading).

- Often does not seem to listen when spoken to directly (e.g., mind seems elsewhere, even in the absence of any obvious distraction).

- Often does not follow through on instructions and fails to finish schoolwork, chores, or duties in the workplace (e.g., starts tasks but quickly loses focus and is easily side tracked).

- Often has difficulty organising tasks and activities (e.g., difficulty managing sequential tasks; difficulty keeping materials and belongings in order; messy, disorganised work; has poor time management; fails to meet deadlines).

- Often avoids, dislikes, or is reluctant to engage in tasks that require sustained mental effort (e.g., schoolwork or homework; for older adolescents and adults, preparing reports, completing forms, reviewing lengthy papers).

- Often loses things necessary for tasks or activities (e.g., school materials, pencils, books, tools, wallets, keys, paperwork, eyeglasses, and mobile telephones).

- Is often easily distracted by extraneous stimuli (for older adolescents and adults, may include unrelated thoughts).

- Is often forgetful in daily activities (e.g., doing chores, running errands; for older adolescents and adults, returning calls, paying bills, keeping appointments).

**Hyperactivity:**

- Often fidgets with or taps hands or feet or squirms in their seat.

- Often leaves a seat in situations when remaining seated is expected (e.g., leaves his or her place in the classroom, in the office or other workplace, or in other situations that require remaining in place).

- Often runs about or climbs in situations where it is inappropriate.

- Often unable to play or engage in leisure activities quietly.

- Is often “on the go,” acting as if “driven by a motor” (e.g., is unable to be or uncomfortable being still for extended time, as in restaurants, meetings; may be experienced by others as being restless or difficult to keep up with).

- Often talks excessively.

**Impulsivity:**

- Often blurts out an answer before a question has been completed (e.g., completes people’s sentences; cannot wait for turn in conversation).

- Often has difficulty waiting his or her turn (e.g., while waiting in line).

- Often interrupts or intrudes on others (e.g., butts into conversations, games, or activities; may start using other people’s things without asking or receiving permission; for adolescents and adults, may intrude into or take over what others are doing).

**Section C: Medical History and Comorbidities**

1. **Comorbidities psychiatric conditions:**

- Depressive disorder

- Anxiety disorder

- Oppositional defiant disorder

- Conduct Disorder

- Others (Please Specify): _____________

- None

2. **Any pharmacotherapy received prior to ADHD treatment?**

- Yes. Please state: _______________

- No

3. **Any non-pharmacotherapy received prior to ADHD treatment?**

- Yes

- No

4. **Duration of the treatment prior to ADHD treatment (months): _____________________**

**BASELINE**

**Section D: ADHD Treatment.**

**1.	Baseline date first seen at current hospital: ______________________**

**2. Facility characteristic at current hospital:**

	In-house OT

	Visiting OT

	Clinical Psychologist

**3**. **Any treatment started at baseline:**

- Yes

- No

**4.**	**If yes, what types of ADHD treatment started?**

	Non-pharmacological treatment (Psychoeducation, psychological treatment, occupational therapy, etc.)

	Pharmacological treatment (stimulant, non-stimulant or others)

	Both pharmacological and non-pharmacological treatment

**5.**	**If taking pharmacotherapy, please state the medication:**

_________________________________________

**5.1** **Dose of medication at initial:** _____________

**5.2 Frequency of medication at initial:**

	Once daily

	More than one daily

**Section E: Baseline CGI rating prior to treatment**

Instruction: Please circle the CGI-Rating score based on the indicator as below.

| **Domains** | **CGI-Rating score** | **Indicator** |
| --- | --- | --- |
| **Academic performance** | 1 | Very much improvement |
|  | 2 | Much improvement |
|  | 3 | Minimal improvement |
|  | 4 | No change |
|  | 5 | Minimally worse |
|  | 6 | Much worse |
|  | 7 | Very much worse |
| **ADHD symptoms** | 1 | Very much improvement |
|  | 2 | Much improvement |
|  | 3 | Minimal improvement |
|  | 4 | No change |
|  | 5 | Minimally worse |
|  | 6 | Much worse |
|  | 7 | Very much worse |
| **Behaviour** | 1 | Very much improvement |
|  | 2 | Much improvement |
|  | 3 | Minimal improvement |
|  | 4 | No change |
|  | 5 | Minimally worse |
|  | 6 | Much worse |
|  | 7 | Very much worse |

**6 MONTHS: FOLLOW UP**

**Section D: ADHD Treatment.**

**1. Date of 6 months review at current hospital: ______________________**

**2. Types of ADHD treatment received:**

- Non-pharmacological treatment (Psychoeducation, psychological treatment, occupational therapy, etc.)

- Pharmacological treatment (stimulant, non-stimulant or others)

- Both of pharmacological and non-pharmacological treatment

- None

**3. Are there any treatment changes?**

- Yes

- No (*If no, skip question 4 to 7 and proceed to Section E)

**4. If yes, state the date of treatment changes: ____________________________**

**5. If there are treatment changes, what type of treatment changes received?**

- Non-pharmacological treatment (Psychoeducation, psychological treatment, occupational therapy, etc.)

- Treatment switch for pharmacological treatment

- Treatment adds on during pharmacological treatment

- Treatment drops during pharmacological treatment

- Treatment discontinuation

**6. Reasons for treatment changes:**

- To reduce adverse effect

- Looking for new alternative treatment

- Risk of abuse

- Suboptimal response

- Costly

- Others (please specify): _________________ _______________

**7.** **If taking pharmacotherapy,** **please state the medication: ________________**

**7.1 Dose of medication at initial: _____________**

**7.2 Frequency of medication at initial:**

- Once daily

- More than one daily

**7.3 Medication name and dose of medication (changes):­­­­­­­­­­­­­­­_________________**

**7.4 Frequency of medication (changes):**

- Once daily

- More than once daily

**Section E: Monitoring Treatment Adverse Effect**

1. **Any side adverse effect from previous ADHD treatment:**

- Drowsiness

- Nausea

- Vomiting

- Decrease appetite and weight loss

- Linear growth impairment

- Insomnia

- Palpitation

- Tics

- Seizures

- Worsening behaviour

- Others (please specify):______________

- No adverse effect / Taking non-pharmacological treatment

2. **Ongoing treatment:**

- Yes

- No (Reason: _______________________

**Section F: Treatment Outcomes**

Instruction: Please circle the CGI-Rating score based on the indicator as below.

| **Domains** | **CGI-Rating score** | **Indicator** |
| --- | --- | --- |
| **Academic performance** | 1 | Very much improvement |
|  | 2 | Much improvement |
|  | 3 | Minimal improvement |
|  | 4 | No change |
|  | 5 | Minimally worse |
|  | 6 | Much worse |
|  | 7 | Very much worse |
| **ADHD symptoms** | 1 | Very much improvement |
|  | 2 | Much improvement |
|  | 3 | Minimal improvement |
|  | 4 | No change |
|  | 5 | Minimally worse |
|  | 6 | Much worse |
|  | 7 | Very much worse |
| **Behaviour** | 1 | Very much improvement |
|  | 2 | Much improvement |
|  | 3 | Minimal improvement |
|  | 4 | No change |
|  | 5 | Minimally worse |
|  | 6 | Much worse |
|  | 7 | Very much worse |

**12 MONTHS FOLLOW UP**

**Section D: ADHD Treatment.**

1. **Date of 12 months review at current hospital: ______________________**

**2. Types of ADHD treatment received:**

- Non-pharmacological treatment (Psychoeducation, psychological treatment, occupational therapy, etc.)

- Pharmacological treatment (stimulant, non-stimulant or others)

- Both of pharmacological and non-pharmacological treatment

- None

**3. Are there any treatment changes?**

- Yes

- No (*If no, skip question 4 to 7 and proceed to Section E)

**4. If yes, state the date of treatment changes: ____________________________**

**5. If there are treatment changes, what type of treatment changes received?**

- Non-pharmacological treatment (Psychoeducation, psychological treatment, occupational therapy, etc.)

- Treatment switch for pharmacological treatment

- Treatment adds on during pharmacological treatment

- Treatment drops during pharmacological treatment

- Treatment discontinuation

**6. Reasons for treatment changes:**

- To reduce adverse effect

- Looking for new alternative treatment

- Risk of abuse

- Suboptimal response

- Costly

- Others (please specify): _________________ _______________

**7.** **If taking pharmacotherapy,** **please state the medication: ________________**

**7.1 Dose of medication at initial: _____________**

**7.2 Frequency of medication at initial:**

- Once daily

- More than one daily

**7.3 Medication name and dose of medication (changes):­­­­­­­­­­­­­­­_________________**

**7.4 Frequency of medication (changes):**

- Once daily

- More than once daily

**Section E: MonitoringTreatment Adverse Effect**

3. **Any side adverse effect from previous ADHD treatment:**

- Drowsiness

- Nausea

- Vomiting

- Decrease appetite and weight loss

- Linear growth impairment

- Insomnia

- Palpitation

- Tics

- Seizures

- Worsening behaviour

- Others (please specify):______________

- No adverse effect / Taking non-pharmacological treatment

4. **Ongoing treatment:**

- Yes

- No (Reason: _______________________

**Section F: Treatment Outcomes**

Instruction: Please circle the CGI-Rating score based on the indicator as below.

| **Domains** | **CGI-Rating score** | **Indicator** |
| --- | --- | --- |
| **Academic performance** | 1 | Very much improvement |
|  | 2 | Much improvement |
|  | 3 | Minimal improvement |
|  | 4 | No change |
|  | 5 | Minimally worse |
|  | 6 | Much worse |
|  | 7 | Very much worse |
| **ADHD symptoms** | 1 | Very much improvement |
|  | 2 | Much improvement |
|  | 3 | Minimal improvement |
|  | 4 | No change |
|  | 5 | Minimally worse |
|  | 6 | Much worse |
|  | 7 | Very much worse |
| **Behaviour** | 1 | Very much improvement |
|  | 2 | Much improvement |
|  | 3 | Minimal improvement |
|  | 4 | No change |
|  | 5 | Minimally worse |
|  | 6 | Much worse |
|  | 7 | Very much worse |

**18 MONTHS FOLLOW UP**

**Section D: ADHD Treatment.**

1. **Date of 18 months review at current hospital: ______________________**

**2. Types of ADHD treatment received:**

- Non-pharmacological treatment (Psychoeducation, psychological treatment, occupational therapy, etc.)

- Pharmacological treatment (stimulant, non-stimulant or others)

- Both of pharmacological and non-pharmacological treatment

- None

**3. Are there any treatment changes?**

- Yes

- No (*If no, skip question 4 to 7 and proceed to Section E)

**4. If yes, state the date of treatment changes: ____________________________**

**5. If there are treatment changes, what type of treatment changes received?**

- Non-pharmacological treatment (Psychoeducation, psychological treatment, occupational therapy, etc.)

- Treatment switch for pharmacological treatment

- Treatment adds on during pharmacological treatment

- Treatment drops during pharmacological treatment

- Treatment discontinuation

**6. Reasons for treatment changes:**

- To reduce adverse effect

- Looking for new alternative treatment

- Risk of abuse

- Suboptimal response

- Costly

- Others (please specify): _________________ _______________

**7.** **If taking pharmacotherapy,** **please state the medication: ________________**

**7.1 Dose of medication at initial: _____________**

**7.2 Frequency of medication at initial:**

- Once daily

- More than one daily

**7.3 Medication name and dose of medication (changes):­­­­­­­­­­­­­­­_________________**

**7.4 Frequency of medication (changes):**

- Once daily

- More than once daily

**Section E: Monitoring Treatment Adverse Effect**

5. **Any side adverse effect from previous ADHD treatment:**

- Drowsiness

- Nausea

- Vomiting

- Decrease appetite and weight loss

- Linear growth impairment

- Insomnia

- Palpitation

- Tics

- Seizures

- Worsening behaviour

- Others (please specify):______________

- No adverse effect / Taking non-pharmacological treatment

6. **Ongoing treatment:**

- Yes

- No (Reason: _______________________

**Section F: Treatment Outcomes**

Instruction: Please circle the CGI-Rating score based on the indicator as below.

| **Domains** | **CGI-Rating score** | **Indicator** |
| --- | --- | --- |
| **Academic performance** | 1 | Very much improvement |
|  | 2 | Much improvement |
|  | 3 | Minimal improvement |
|  | 4 | No change |
|  | 5 | Minimally worse |
|  | 6 | Much worse |
|  | 7 | Very much worse |
| **ADHD symptoms** | 1 | Very much improvement |
|  | 2 | Much improvement |
|  | 3 | Minimal improvement |
|  | 4 | No change |
|  | 5 | Minimally worse |
|  | 6 | Much worse |
|  | 7 | Very much worse |
| **Behaviour** | 1 | Very much improvement |
|  | 2 | Much improvement |
|  | 3 | Minimal improvement |
|  | 4 | No change |
|  | 5 | Minimally worse |
|  | 6 | Much worse |
|  | 7 | Very much worse |
